# Face, eye, and body selective responses in fusiform gyrus and adjacent cortex: an intracranial EEG study

**DOI:** 10.3389/fnhum.2014.00642

**Published:** 2014-08-21

**Authors:** Andrew D. Engell, Gregory McCarthy

**Affiliations:** ^1^Kenyon Psychological Neuroscience Laboratory, Department of Psychology, Kenyon CollegeGambier, OH, USA; ^2^Human Neuroscience Laboratory, Department of Psychology, Yale UniversityNew Haven, CT, USA

**Keywords:** face area, body area, eye, face-part, N200, ECoG, iEEG

## Abstract

Functional MRI (fMRI) studies have investigated the degree to which processing of whole faces, face-parts, and bodies are differentially localized within the fusiform gyrus and adjacent ventral occipitotemporal cortex. While some studies have emphasized the spatial differentiation of processing into discrete areas, others have emphasized the overlap of processing and the importance of distributed patterns of activity. Intracranial EEG (iEEG) recorded from subdural electrodes provides excellent temporal and spatial resolution of local neural activity, and thus provides an alternative method to fMRI for studying differences and commonalities in face and body processing. In this study we recorded iEEG from 12 patients while they viewed images of novel faces, isolated eyes, headless bodies, and flowers. Event-related potential analysis identified 69 occipitotemporal sites at which there was a face-, eye-, or body-selective response when contrasted to flowers. However, when comparing faces, eyes, and bodies to each other at these sites, we identified only 3 face-specific, 13 eye-specific, and 1 body-specific electrodes. Thus, at the majority of sites, faces, eyes, and bodies evoked similar responses. However, we identified ten locations at which the amplitude of the responses spatially varied across adjacent electrodes, indicating that the configuration of current sources and sinks were different for faces, eyes, and bodies. Our results also demonstrate that eye-sensitive regions are more abundant and more purely selective than face- or body-sensitive regions, particularly in lateral occipitotemporal cortex.

## Introduction

The discovery of face-selective neurons in the macaque temporal lobe (Gross et al., 1969, 1972) set in motion a productive research program in the study of face perception in the primate visual system. Functional neuroimaging (Sergent et al., 1992; Haxby et al., 1994; Puce et al., 1995) and intracranial EEG (iEEG) (Allison et al., 1994a,b) studies expanded that program to the neural basis of face perception in the human. These early studies showed that faces selectively activated regions of the (predominantly right) ventral occipitotemporal cortex (VOTC). In particular, functional MRI studies have consistently identified a small region of the lateral mid-fusiform gyrus as face selective, a region that is often referred to as the “fusiform face area” (FFA; Kanwisher et al., 1997). Subsequent fMRI research identified a region posterior to the FFA, dubbed the “occipital face area” (OFA) that is also preferentially activated by the perception of faces (Gauthier et al., 2000). These regions have since been promoted as “core nodes” in an extended face processing network (e.g., Haxby et al., 2000; Rossion et al., 2003; Calder and Young, 2005; Ishai, 2008; Pitcher et al., 2011).

While regions within the VOTC are unequivocally sensitive to faces, VOTC regions are also active during perception of non-face corporeal stimuli. For example, the perception of bodies also evokes a larger hemodynamic response than the perception of non-corporeal objects along part of the fusiform gyrus (Schwarzlose et al., 2005; Peelen and Downing, 2005; Peelen et al., 2006; Pinsk et al., 2009; van de Riet et al., 2009; see de Gelder et al., 2010). Although there is substantial overlap between VOTC areas activated by faces and by bodies, some studies have identified a discrete region activated by bodies that is dissociable from the FFA and which has been named the “fusiform body area” (FBA; Schwarzlose et al., 2005). Isolated bodies also activate lateral occipitotemporal cortex (LOTC) at the intersection of the anterior occipital and inferior temporal gyri, an area named the “extrastriate body area” (EBA; Downing and Peelen, 2011).

Other studies have shown that regions of the occipitotemporal cortex (OTC) co-extensive with the FFA, the FBA, and the EBA respond to biological motion (Bonda et al., 1996; Grossman and Blake, 2002; Peelen et al., 2006; Grezes et al., 2007; Pichon et al., 2008; Engell and McCarthy, 2013; Shultz et al., 2014). These results suggest the possibility that these regions are not responding to specific body parts *per se*, but are engaged by the processing of intentional or social agents (Shultz et al., 2014). Indeed, in a recent study, Shultz and McCarthy (Shultz and McCarthy, 2012) showed that areas of the VOTC co-extensive with the FFA responded to the apparently purposeful motion of machines.

Most studies of non-face corporeal perception have been conducted using fMRI. Intracranial electroencephalography has millisecond temporal resolution and, depending upon the configuration of electrodes, can have high anatomical resolution (although coverage can be sporadic). There have been very few iEEG studies of social agent perception that have focused on stimuli other than whole-faces. One of the few such reports found that images of hands evoked a category-selective event-related potential (ERP) from recording sites on the right VOTC and left LOTC cortices (McCarthy et al., 1999). At these locations there was no concomitant category-selective response to faces or face-parts (eyes, lips, and noses). Similarly, a more recent report found a single body-selective site on the right LOTC at which there was no appreciable response to faces (Pourtois et al., 2007).

These findings provide limited support for the idea that face and non-face body parts are processed in distinct brain areas. However, given the extent of the activation overlap between faces and bodies observed in fMRI, a more systematic study is warranted, particularly since one of the two aforementioned studies used images of isolated hands rather than bodies as a stimulus (McCarthy et al., 1999). Subsequent neuroimaging has shown that hands and bodies evoke dissociable neural responses (Bracci et al., 2010). The second study reported results from a single electrode within a single patient. Small samples are common in iEEG as this method relies on the participation of individuals undergoing invasive brain procedures, often for pharmacologically intractable epilepsy. Nonetheless, results from a single electrode raise concerns about the replicability and generalizability of the findings.

Here we use iEEG to investigate the functional selectivity and spatial relationship of the response to three visual categories of social agents (whole faces, eyes in isolation, and headless-bodies). Both time-locked ERPs and event-related spectral perturbations (ERSPs) were investigated. We address the possible limitations of previous iEEG experiments by using images of whole bodies (without heads) rather than isolated body parts, and by using a large sample of 1536 electrode sites across twelve patients. In addition to evaluating the amplitude of the ERP at each subdural electrode to faces, bodies, and eyes, we also examined the spatial distribution of the ERPs evoked by each stimulus type across adjacent electrodes. The spatial configuration of current sinks and sources relative to the recording electrodes determines the spatial distribution of voltage over the cortex. If the same source configuration was responsible for the ERP evoked by each different stimulus, then the spatial distribution would be the same. However, if the spatial distribution of voltage evoked by faces, bodies, and eyes differed across closely adjacent sites, this would be strong evidence that a different pattern of neural activity, and perhaps a different subset of neurons, was activated by the different stimulus categories.

## Materials and methods

### Procedure

**Figure 1:**
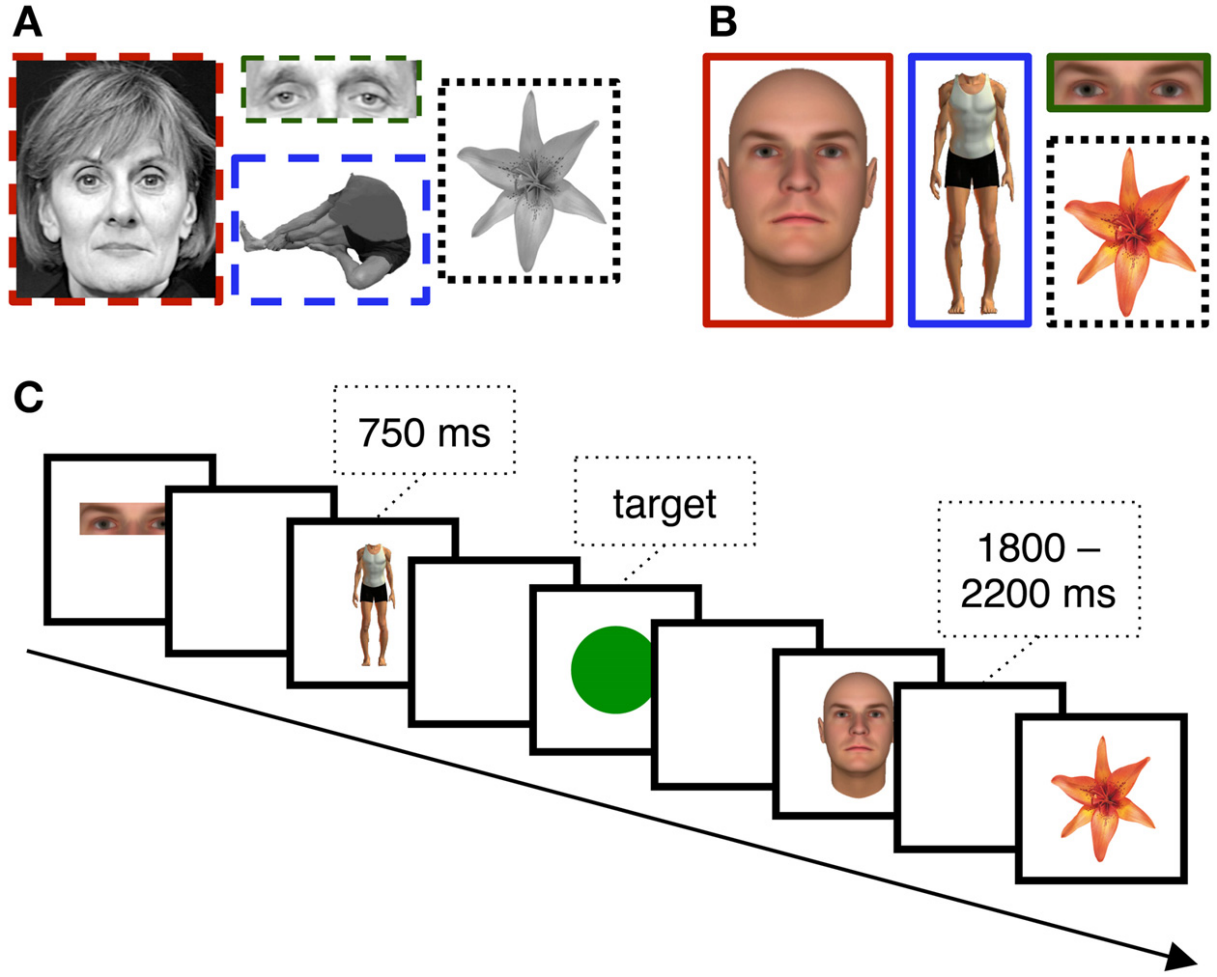
**EEG Stimuli and Task**. Example **(A)** grayscale and **(B)** color stimuli used for different subsets of the patients. The task **(C)** was simple target detection task in which patients reported the presence of the circle targets with a key-press.

Stimulus presentation was computer controlled and displayed on a 17″ LCD monitor (800 × 600 pixels) positioned on a table over the patient's bed. The viewing distance was adjusted for patient comfort. Patients were asked to view sequentially presented stimuli that were randomly selected from four categories: novel faces, eyes in isolation, headless bodies, and flowers (Figures 1A,B). In total, patients viewed 40 unique exemplars from each stimulus category. Each image was displayed for 750 ms with a jittered stimulus onset asynchrony that varied randomly between 1800 and 2200 ms (Figure 1C). In the first version of the experiment (see below) images from the face, eye, body, and flower categories were presented a roughly equal number of times (77–83 trials of each for patient 1, and 174–188 trials of each for patient 2). In the second version, 40 trials were presented from each of these categories. Two patients experienced a longer version in which 80 trials were presented from each category. To ensure the patient's engagement with the task, a target circle was presented on ~11.1% of trials to which a speeded button press response was required. Presentation of the stimuli was intermittently paused to give the patients a rest period.

### Stimuli

The first two of our twelve participants saw grayscale images (Figure 1A). Face stimuli were created from photographs taken from various sources (see Allison et al., 1999 for details). Eye stimuli were the same pictures used for faces, but cropped so that only the eye-region remained visible. Body stimuli were photographs of males and females, digitally cropped so that the head was removed. Flower stimuli were color photographs converted to grayscale images. Prior studies from our laboratory have shown that face-selective ERPs evoked at subdural VOTC electrodes are readily dissociable from many different non-corporeal object categories, including letter strings, patterns, and flowers (cf. Allison et al., 1994a, 1999; McCarthy et al., 1999; Engell and McCarthy, 2010, 2011). We chose flowers as control stimuli because they are a category of living stimuli with visual symmetry and can be readily individualized.

The remaining ten participants saw color stimuli (Figure 1B). Face stimuli were realistic faces created using FaceGen (Singular Inversions, Toronto, Ontario, Canada). Eye stimuli were the same images used for faces, but cropped so that only the eye-region remained visible. Body stimuli were created using Poser 6.0 (Curious Labs Inc., Santa Cruz, California, USA). Flower stimuli were the same as those described above, but were not converted to grayscale.

### EEG acquisition

Recordings were obtained from 1536 electrodes implanted in 12 patients (median age = 33 years, age range: 18–54 years, 8 female, 4 male) with medically intractable epilepsy who were being evaluated for possible surgery by the Yale Epilepsy Surgery Program (Spencer et al., 1982). In these patients, strips or grids of stainless steel electrodes (2.2 mm surface diameter) were placed subdurally on the cortical surface. The placement of the strips was determined by the clinical needs of each patient, and thus electrode locations varied across individuals. The studies reported here were included among other sensory and cognitive experiments in which each subject participated, typically 4–8 days following implantation of electrodes. At the time of participation, medication levels to control seizures and post-operative pain varied across patients. The EEG experiments were not conducted immediately before or after seizures nor were any of our sites of interest revealed to be in epileptogenic cortex. The EEG protocol was approved by the IRB of the Yale University School of Medicine. All participants provided informed consent.

Local field potentials were recorded and amplified with a common reference using an SA Instruments EEG amplifier system with a 0.1–100 Hz bandpass. The reference was a small post implanted in the outer table of the patient's skull. The location of this post varied across patients, but it was always in the skull adjacent to superior frontal or parietal cortex. Most often, the post was implanted at the top of the skull in a region roughly adjacent to electrodes C3 or C4 of the 10-20 EEG Electrode system. From each patient we simultaneously recorded from 128 electrodes with a concentration of sites on ventral occipitotemporal, lateral occipital, posterior lateral temporal, and parietal cortices. The EEG signal was continuously digitized with 14-bit resolution and a sampling rate of 500 Hz using a Microstar 4200 A/D data acquisition board. The digitized signal was written to disk using a custom PC-based acquisition system. A digital code unique to each experimental condition was recorded in a separate channel at the onset of each stimulus presentation.

### Electrode localization

**Figure 2:**
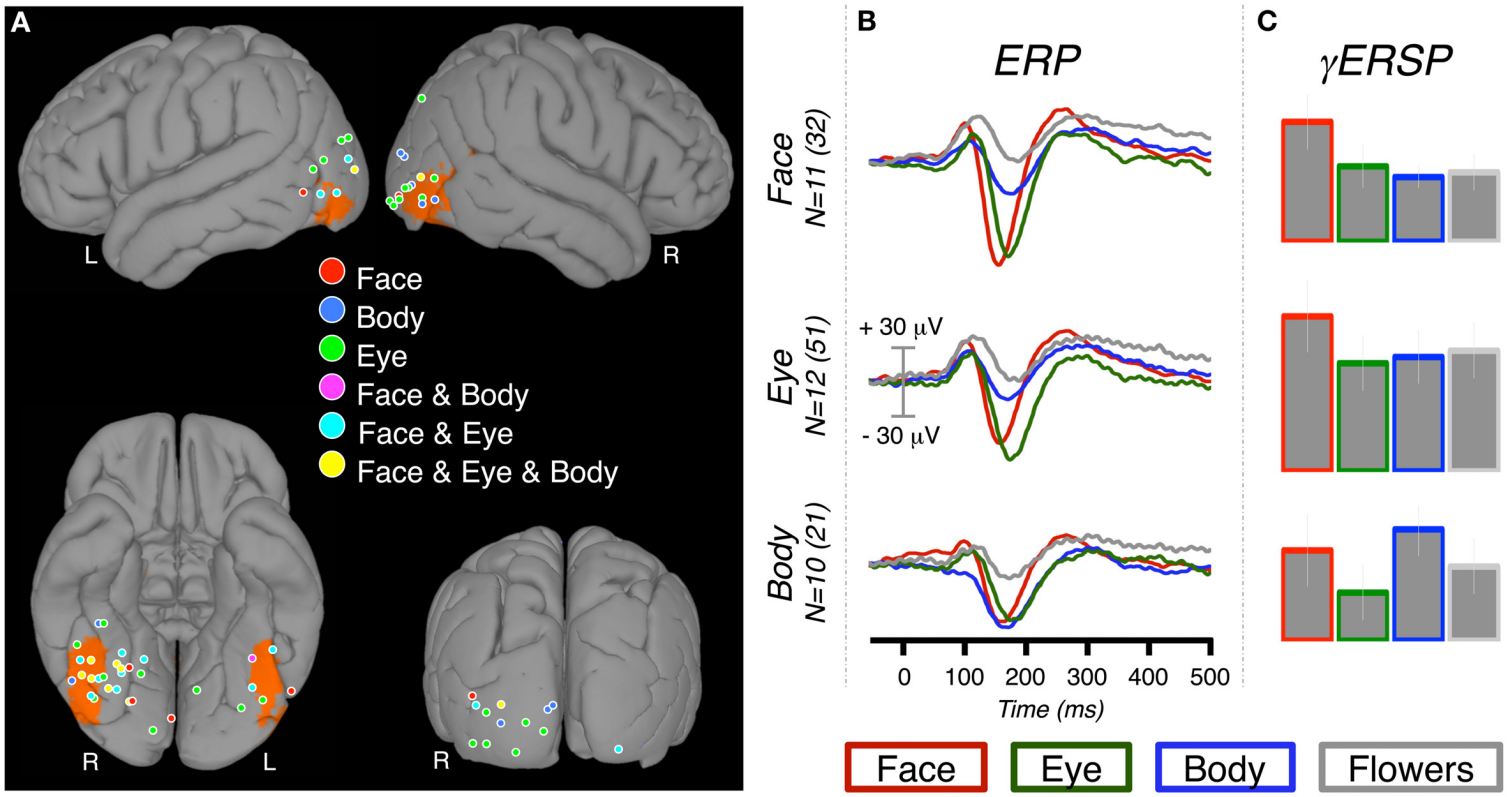
**ERP and γERSP response at category-selective sites. (A)** The locations of 69 category-selective electrodes displayed on a standard brain (left panel). Selectivity was defined by contrasting each of the conditions of interest (faces, eyes, bodies) to flowers. Therefore, a single electrode could be identified as “selective” for more than one condition. The color at each location indicates which category (or categories) met selectivity criteria (see Materials and Methods). For reference to standard imaging results, the ORANGE overlay indicates voxels at which there is a ≥33% probability of being face-selective in a face vs. scene fMRI experiment (Engell and McCarthy, 2013). **(B)** The grand-average ERPs for each condition were calculated at all electrodes that were category-selective for one or more conditions. The grand-averages can therefore include sites that were selective for multiple conditions. For instance, the body-selective ERP (bottom row) includes the response recorded from the “Body,” “Face & Body,” and “Face & Eye & Body” locations. The grand average ERP was created by averaging patient ERPs, which could each include one or more electrodes. We report both the patient sample size (*N*) and the total number of electrodes. **(C)** The relative increase in event-related gamma power at the same category-selective sites.

**Figure 3:**
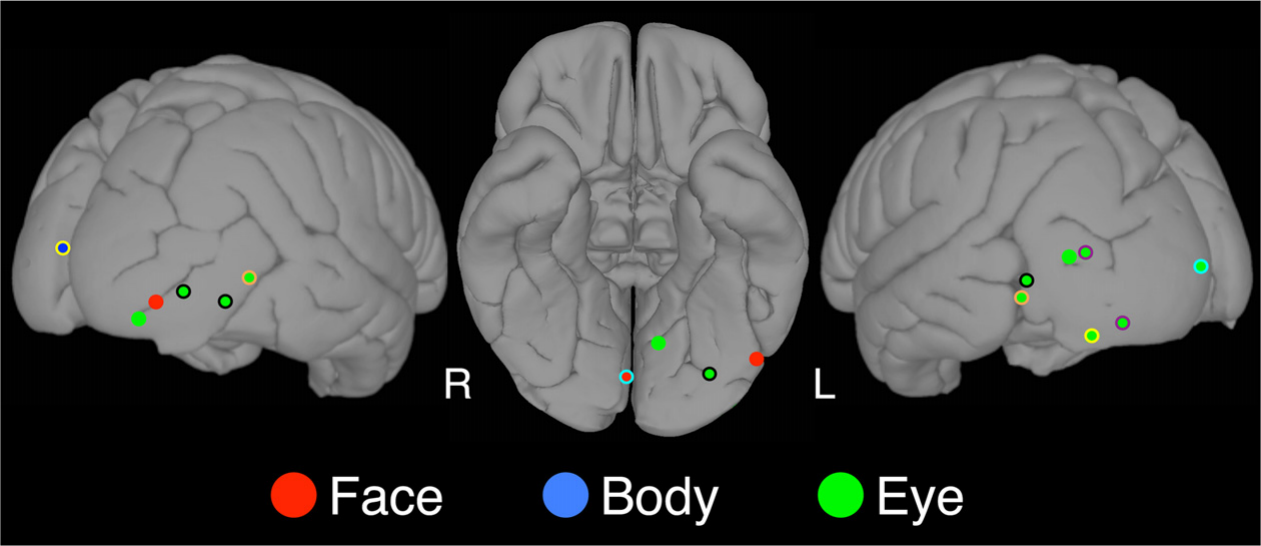
**Category-specific sites**. The locations of category-specific electrodes overlaid on a standard brain. At these sites, the category-selectivity was determined by contrasting the condition of interest to *all* other conditions, not only flowers. Using this more conservative criterion we found three face-selective, thirteen eye-selective, and one body-selective site. A color border indicates electrodes that were contributed by the same patient. For instance, the orange border around the eye selective sites on the left and the right occipitotemporal cortex indicates that the same patient contributed these two electrodes.

A high-resolution anatomical scan (1 × 1 × 1.5 mm) was acquired for each patient prior to implantation. Post-implant CT scans in which the electrodes were easily detected and localized in 3D were then co-registered to the anatomical MR data. Each patient's brain was transformed to MNI space using the Bioimage Suite software package (http://www.bioimagesuite.org) to facilitate visualization of recording sites of interest from all patients on a standard brain. In cases in which the inherent imprecision of spatial normalization resulted in an electrode appearing just off the brain, the electrode position was projected to the cortical surface. This approach allowed for a convenient graphical representation of the overall distribution of electrodes on the brain's surface (Figures 2A, 3). However, as the exact gyral and sulcal boundaries of the brain varied among our subjects, this summary view does not precisely reflect the location of any individual electrode with respect to anatomical landmarks of the subject's brain in which it was located.

**Figure 4:**
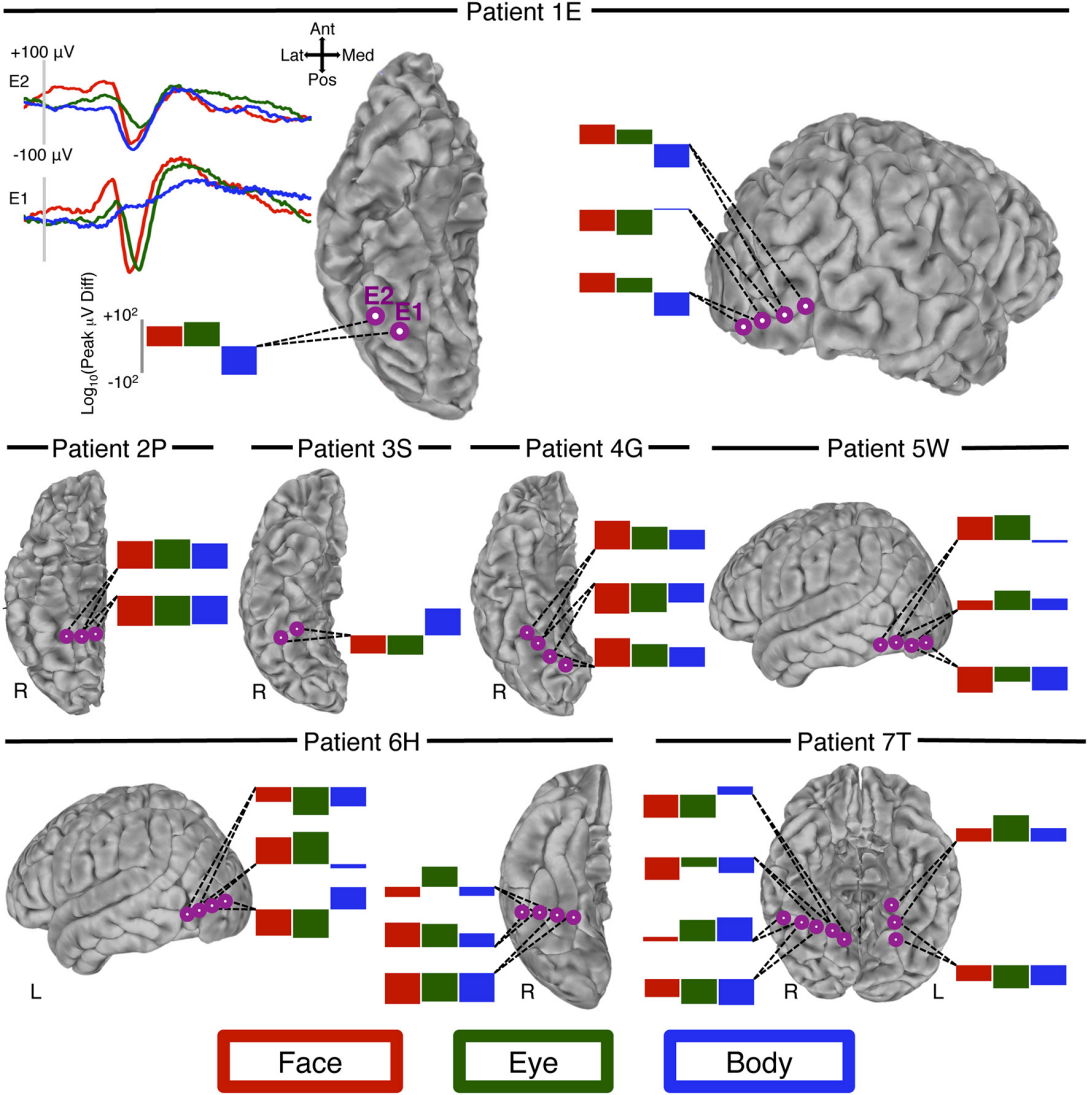
**Peak-voltage changes over space**. We visually identified 12 locations (seven patients) at which adjacent electrodes showed an N200 to at least two of our conditions of interest (faces, eyes, bodies), and at which the peak-voltage to these conditions changed at different rates over space. For patient 1E (top row, left column) we display the waveform from two adjacent electrodes on the right ventral temporal surface. Inspection of these waveforms shows that at electrode E1 there is a prominent N200 to both faces and eyes, but not bodies. At the adjacent electrode, E2, bodies evoke an N200 that is qualitatively larger than faces and eyes. Moreover, compared to the response at E1 the eye-N200 has diminished more sharply than the face-N200. The log of the peak difference is displayed in the bar graph. For all other patients we display only these bar graphs to represent the rate of change for each of the three conditions. At each location (i.e., each collection of adjacent sites) two or more of the conditions experience a different rate of change over space, indicating differing patterns of current sinks and sources.

Instances of conditional voltage changes over space (see Rate of Voltage Changes Over Space) are also displayed on cortical surfaces. In an effort to preserve the relationship of electrode locations to sulcal and gyral boundaries, we projected these electrodes on to each individual's brain (Figure 4, P1, P2, P3, P4, & P7). However, for two of the participants the signal to noise was insufficient to achieve quality segmentation so the electrodes from these individuals are shown on a canonical brain surface (Figure 4, P5 & P6).

### Event-related Potential (ERP) analysis

ERP analyses were performed using custom MATLAB (The Mathworks, Inc.) functions. Residual line noise (60 Hz) filtering was performed in Matlab using a 5th order Butterworth filter that was applied in a temporally symmetric manner to avoid introducing phase shifts. Baseline adjusted ERPs were created by signal averaging the EEG across trials for each experimental condition and subtracting from each time-point the average of a 100 ms pre-stimulus epoch. Low-pass filtering was achieved with a temporally symmetric smoothing kernel with a total length of five time-points (from −2 to +2 time points) that was convolved with the average ERP waveforms prior to amplitude and latency measurements of the N200.

A computer algorithm was used to identify electrodes that were “selective” for a particular category. Guided by previously published criteria (Allison et al., 1999), face-, eye-, and body-selective sites were defined as those with a peak negativity occurring between 160 and 240 ms post stimulus onset (N200) that was at least −50 μ V in amplitude and at least twice as large for the category of interest than for the control condition (flowers). Similar selection criterion (i.e., a category response twice as large as for all other tested categories) has previously been used in both single cell (e.g., Perrett et al., 1982; Baylis et al., 1985; Leonard et al., 1985) and human local field potential (LFP) (e.g., Puce et al., 1997; Allison et al., 1999; McCarthy et al., 1999; Puce et al., 1999; Engell and McCarthy, 2010, 2011) investigations of face-selective responses. Consistent with previous human LFP studies, this was based on the qualitative comparison of the peak magnitude of ERPs. Automated detection by the computer algorithm was followed by visual inspection by the authors to screen for artifacts. Nine of 41 face-selective electrodes, three of 54 eye-selective electrodes, and seven of 29 body-selective electrodes that were identified by the computer algorithm were excluded from analysis.

For each set of category-selective electrodes, we created an average ERP from all electrodes contributed by a given patient. We then identified the peak amplitude of each category-evoked response from within our epoch of interest (160–240 ms) and the latency at which the peak occurred. Wilcoxon signed rank tests were then used for pairwise contrasts of the four conditions to test for differences in the peak amplitude and latency of the N200 response. For each group of category-selective electrodes, we performed five pairwise tests, which included all possible pairings except for the category-selective condition vs. flowers. The latter test was not performed because category vs. flower was our selection criteria. We used a Bonferroni correction to adjust the significance threshold for our five contrasts from *p* < 0.05 to *p* < 0.01.

The normalized locations (MNI) of the category-selective electrodes were plotted onto a standard brain (Figure 2A). K-means clustering, as implemented with the “kmeans” function in MATLAB′s (The Mathworks, Inc.) Statistics Toolbox, was used to further summarize the electrode locations by segmenting them into four clusters and identifying the locations of the cluster centroids. We chose *k* = 4 because visual inspection of the electrode locations suggested a cluster on the ventral temporal surface, and another on the occipitotemporal surface of each hemisphere. The selection of four clusters was supported by the quantitative observation that four clusters explained 89, 85, and 87% of the total spatial variance for faces, eyes, and bodies, respectively.

To test for latency differences we identified the peak of the N200 (minimum amplitude) within our critical time window (160–240 ms) for each electrode. The latencies of these peaks for each condition were then contrasted using Wilcoxon signed rank tests.

Finally, we identified category-*specific* electrodes. At these sites the response to a given category (faces, eyes, or bodies) met the criteria for selectivity as compared to *all* other corporeal categories and flowers.

### Rate of voltage changes over space

The changes in voltage created by the current sinks and sources of active neurons can be recorded throughout the volume conductor of the brain, but the strength of the voltage diminishes with distance from the source, resulting in a weaker signal at more distal recording sites. The rate at which this signal decays (as a percentage of the peak amplitude) should be the same for ERPs generated by the same configuration of current sinks and sources; this is true regardless of the initial strength of the signal. If ERPs evoked by two different stimulus categories fall off at different rates across adjacent electrodes it indicates a different configuration of sinks and sources, and thus a different pattern of neural activity. We looked for all instances of differential voltage changes over space in the peak ERP response to faces, eyes, and bodies by visually inspecting all ERPs from the 12 patients. At these locations, we quantified the change for each condition by calculating the log of the peak voltage difference between the adjacent electrodes. This returned a complex number for negative peak differences. For these values we report the negative of the real part of the complex number.

### Event-related Spectral Perturbation (ERSP) analysis

In addition to the ERP analysis, we also analyzed the EEG using a time-frequency approach that evaluated event-related changes in gamma power (gamma event-related spectral perturbations; γERSP) at each of the category-selective sites. Following our prior reports (Engell and McCarthy, 2010, 2011, 2014) we removed the mean signal-averaged ERP from the raw EEG signal for each trial prior to ERSP analysis. This ensured that any significant spectral differences between categories did not merely reflect the frequency composition of the phase-locked ERP. As a result of this approach, the frequency-domain analysis reported here is insensitive to spectral changes that undergo phase resetting (i.e., phase-locked “evoked” EEG responses). However, these signal components are well captured in the time-domain analysis (i.e., ERP), resulting in a full characterization of the data.

Event-related spectral perturbations were computed using EEGLAB v7.1 (Delorme and Makeig, 2004) and MATLAB v7.9 (The Mathworks, Inc.). Time-frequency power spectra were estimated using Morlet wavelet analysis based on 3 cycles at the lowest frequency (11.6 Hz) increasing to 16 cycles at the highest frequency (125 Hz). Change in power induced by each category (i.e., ERSP) was estimated by calculating the ratio of log power (dB) between the post-stimulus and pre-stimulus epochs. ERSPs within the gamma band (30–100 Hz) were averaged at each time-point to create a “gamma power-wave” over time. This frequency range for gamma was selected on the basis of the prior literature. Reports in the animal (Singer and Gray, 1995; Tallon-Baudry and Bertrand, 1999) and human (e.g., Lachaux et al., 2005; Tsuchiya et al., 2008; Fisch et al., 2009; Engell and McCarthy, 2010, 2011, 2014; Engell et al., 2012) literatures have defined 30 Hz as the lower bound of the gamma band. These same human intracranial studies have reported an upper bound for gamma between 70 and 200 Hz. The amplifiers used in our studies imposed a 100 Hz (−3db) upper limit on the iEEG signal, so we restricted the upper range of the gamma band to 100 Hz. For each condition and each site with a category-selective N200 we estimated the area under curve (AUC) within an epoch that appeared to be most sensitive to the task (Engell and McCarthy, 2011, 2014; Engell et al., 2012). Across conditions, 150–600 ms showed the largest changes in gamma power for all conditions and we therefore focused our analysis on this window. Where appropriate these AUC estimates were contrasted with paired-sample *t*-tests.

## Results

### Event-related potentials

#### Face-selective electrodes

We identified 32 face-selective electrodes (20 RH, 12 LH) across 11 patients (Table 1, Figure 2). At these locations, the peak amplitude medians of faces, eyes, bodies, and flowers were −106.66, −94.32, −50.54, and −19.40, respectively. The Wilcoxon signed rank test showed that the face response was larger (i.e., more negative) than the body response, *Z* = 2.76, *p* = 0.006, but not the eye response, *Z* = 1.07, *p* = 0.286. Note that selection of these sites was solely based on the faces > control contrast, so the selection process did not necessitate that faces would be larger than bodies or eyes. The peak response to eyes was larger than bodies, *Z* = 2.93, *p* = 0.003, and flowers, *Z* = 2.93, *p* = 0.003. The peak response to bodies was larger than flowers, *Z* = 2.85, *p* = 0.004.

**Table 1:** **Electrode locations of face-selective sites within each spatial cluster**.

**MNI Coordinates of Face-Selective Electrode Locations (*N* = 32)**											
**Right ventral temporal**			**Right occipitotemporal**			**Left ventral temporal**			**Left occipitotemporal**		
***X***	***Y***	***Z***	***X***	***Y***	***Z***	***X***	***Y***	***Z***	***X***	***Y***	***Z***
*33*	−*55*	−*17*	*27*	−*80*	−*8*	−*46*	−*59*	−*20*	−*38*	−*89*	*1*
45	−49	−26	53	−78	3	−34	−48	−22	−25	−96	8
45	−56	−22	**37**	**−91**	**−6**	−34	−61	−23	−36	−91	8
40	−50	−16	24	−70	−14	−42	−42	−26	−37	−93	7
40	−58	−21	22	−69	−15	−52	−74	−14	−41	−85	2
40	−67	−25	22	−94	−9	**−53**	**−65**	**−20**	−41	−89	−4
37	−58	−20	**4**	**−79**	**−8**	−58	−65	−12	−45	−82	−14
33	−63	−17									
29	−52	−13									
29	−63	−18									
27	−53	−14									
27	−56	−15									
26	−46	−17									
25	−53	−9									
18	−51	−8									

The MNI coordinates from each of the 32 face-selective electrodes are grouped according to which cluster they were assigned to by the k-means clustering analysis. The MNI coordinates in the first row are the centroid locations within each cluster. Coordinates in bold typeface indicate electrodes that were face-specific as well as face-selective (see Materials and Methods).

The latency medians of faces, eyes, bodies, and flowers were 162, 178, 186, and 192 ms, respectively. The Wilcoxon signed rank test showed that the face response was marginally earlier than the eye response, *Z* = 2.54, *p* = 0.011, and significantly earlier than the body response, *Z* = 2.67, *p* = 0.008. The latency of the peak to eyes did not differ from the latency of the peak to bodies, *Z* = 1.11, *p* = 0.266, but was marginally earlier than flowers, *Z* = 2.40, *p* = 0.016. Bodies and flowers did not differ, *Z* = 0.71, *p* = 0.476.

**Figure 5:**
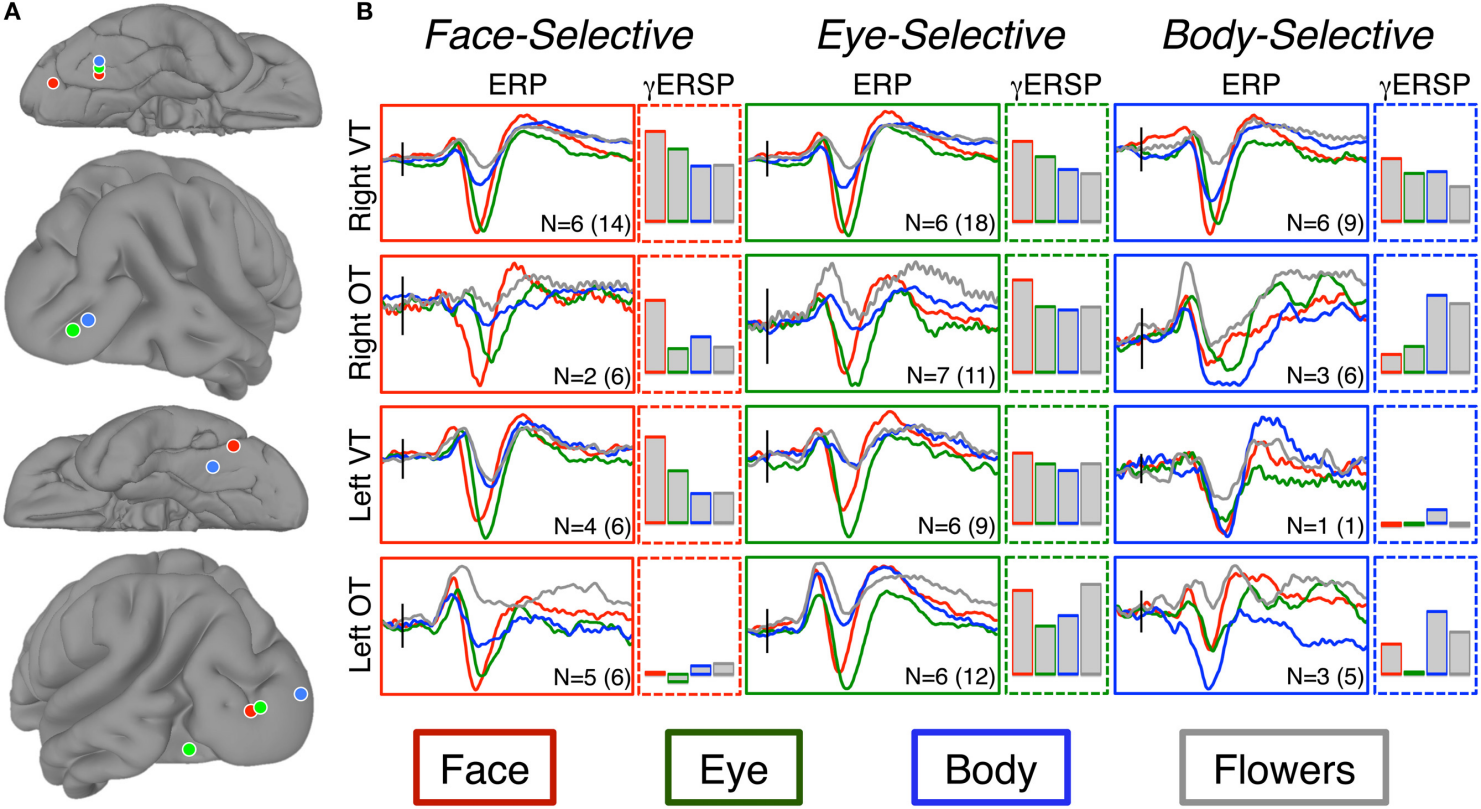
**Results of spatial-clustering. (A)** The locations of the centroids from the k-means cluster analysis as seen on the right ventral tremporal (Right VT; 1st row), right occipitotemporal (Right OT; 2nd row), left ventral temporal (Left VT; 3rd row), and left occipitotemporal (Left OT; 4th row) surfaces. Centroids are displayed for faces (red), eyes (green), and bodies (blue). **(B)** Grand-average ERPs were calculated from the electrodes within each spatial cluster. The “I” bar in each plot represents 50 μ V along the y-axis and is located along the x-axis at the time of stimulus onset. We report both the patient sample size (N) and the total number of electrodes included in each ERP. Bar graphs show the relative increase in event-related gamma power (AUC of log power change from baseline) at these same electrodes. Bar height indicates AUC between 0 and 100 db^2^. Note, only *increases* in gamma power are shown. At the body-selective sites in the left ventral and occipitotemporal regions there was desynchronization to flowers and to eyes, respectively.

The cluster detection algorithm (see Materials and Methods) identified two spatial clusters of electrodes within each hemisphere for face-selective responses. The cluster centroids in the right hemisphere were located at 33, −55, 17 and 27, −80, −8. The cluster centroids in the left hemisphere were located at −46, −59, −20 and −38, −89, 1 (Figure 5). Despite the sparse sampling inherent in iEEG, these centroids roughly corresponded to face-selectivity peaks as identified by the Atlas of Social Agent Perception (Engell and McCarthy, 2013). The small sample sizes within each cluster precluded statistical analysis of the ERPs. We describe the relevant qualitative results from within these clusters in the context of our discussion section.

In a second analysis, we identified face-*specific*, rather than face-*selective* (see Materials and Methods), electrodes. We found three face-specific sites (2 RH, 1 LH) contributed by three patients (bolded coordinates in Table 1).

#### Eye-selective electrodes

We identified 51 eye-selective electrodes (30 RH, 21 LH) across 12 patients (Table 2, Figure 2). At these locations, the peak amplitude medians of faces, eyes, bodies, and flowers were −51.66, −97.79, −27.40, and −15.16 μ V, respectively. The Wilcoxon signed rank test showed that the eye response was larger than the response to faces, *Z* = 2.90, *p* = 0.004, and bodies, *Z* = 3.06, *p* = 0.002. The average peak response to faces was significantly larger than to bodies, *Z* = 2.75, *p* = 0.006, and flowers, *Z* = 3.06, *p* = 0.002. The body response was marginally larger than the flower response, *Z* = 2.20, *p* = 0.028.

**Table 2:** **Electrode locations of eye-selective sites within each spatial cluster**.

**MNI Coordinates of Eye-Selective Electrode Locations (***N*** = **51**)**											
**Right ventral temporal**			**Right occipitotemporal**			**Left ventral temporal**			**Left occipitotemporal**		
***X***	***Y***	***Z***	***X***	***Y***	***Z***	***X***	***Y***	***Z***	***X***	***Y***	***Z***
*33*	−*55*	−*18*	*38*	−*85*	−*2*	−*40*	−*68*	−*12*	−*31*	−*92*	*1*
45	−41	−28	**54**	**−72**	**3**	**−8**	**−64**	**−9**	−9	−98	−1
45	−49	−26	53	−78	3	**−30**	**−73**	**−11**	**−15**	**−98**	**4**
45	−56	−22	**51**	**−77**	**−5**	−34	−61	−23	−18	−98	−9
40	−50	−16	**44**	**−85**	**−4**	−39	−68	−12	−25	−96	8
40	−58	−21	43	−86	−4	−42	−42	−26	**−29**	**−91**	**−11**
40	−67	−25	37	−97	−9	−45	−82	−14	−29	−99	5
40	−67	−25	36	−81	36	−52	−74	−14	**−36**	**−88**	**−13**
37	−58	−20	35	−91	−7	**−53**	**−71**	**−3**	−37	−93	7
35	−57	−19	**32**	**−91**	**−12**	**−55**	**−75**	**1**	−41	−85	2
34	−32	−27	22	−94	−9				−41	−89	−4
33	−63	−17	12	−82	−14				**−43**	**−85**	**11**
29	−52	−13							**−45**	**−84**	**9**
29	−63	−18									
27	−53	−14									
27	−56	−15									
26	−46	−17									
24	−70	−14									
20	−56	−6									
18	−51	−8									

The MNI coordinates from each of the 51 eye-selective electrodes are grouped according to which cluster they were assigned to by the k-means clustering analysis. The MNI coordinates in the first row are the centroid locations within each cluster. Coordinates in bold typeface indicate electrodes that were eye-specific as well as eye-selective (see Materials and Methods).

The latency medians of faces, eyes, bodies, and flowers were 168, 179, 187, and 182 ms, respectively. The Wilcoxon signed rank test showed that the eye response was significantly later than the face response, *Z* = 2.71, *p* = 0.007, but did not differ from the body response, *Z* = 0.67, *p* = 0.505. The latency of the peak to faces was marginally earlier than bodies, *Z* = 1.94, *p* = 0.052, and significantly earlier than flowers, *Z* = 2.59, *p* = 0.010. Bodies and flowers did not differ, *Z* = 1.57, *p* = 0.116.

The cluster detection algorithm identified two spatial clusters of electrodes within each hemisphere for the eye-selective ERPs. The cluster centroids in the right hemisphere were located at 33, −55, 18 and 38, −85, −2. The cluster centroids in the left hemisphere were located at −40, −68, −12 and −31, −92, 1 (Figure 5). The small sample sizes within each cluster precluded statistical analysis of the ERPs. We describe the relevant qualitative results from within these clusters in the context of our discussion section.

In a second analysis, we identified eye-*specific*, rather than eye-*selective* (see Materials and Methods), electrodes. We found 13 eye-specific sites (4 RH, 9 LH) contributed by eight patients (bolded coordinates in Table 2). Within patients from whom several eye-specific sites were identified, there was only one instance in which the electrodes were closely adjacent. In all other cases the electrodes were located in different within-hemisphere locations (e.g., lateral temporal and ventral temporal cortices) or in different hemispheres.

#### Body-selective electrodes

We identified 21 body-selective electrodes (15 RH, 6 LH) across ten patients (Table 3, Figure 2). At these locations, the peak amplitude medians of faces, eyes, bodies, and flowers were −83.63, −69.88, −75.45, and −21.30 μ V, respectively. The Wilcoxon signed rank test showed that the body response was not larger than faces, *Z* = 0.56, *p* = 0.575, or eyes, *Z* = 0.66, *p* = 0.508. The average peak response to faces was significantly larger than to flowers, *Z* = 2.60, *p* = 0.009, but not to eyes, *Z* = 0.05, *p* = 0.959. The eye response was marginally larger than the flower response, *Z* = 2.50, *p* = 0.013.

**Table 3:** **Electrode locations of body-selective sites within each spatial cluster**.

**MNI Coordinates of Body-Selective Electrode Locations (*N* = 21)**											
**Right ventral temporal**			**Right occipitotemporal**			**Left ventral temporal**			**Left occipitotemporal**		
***X***	***Y***	***Z***	***X***	***Y***	***Z***	***X***	***Y***	***Z***	***X***	***Y***	***Z***
*36*	−*55*	−*19*	*45*	−*83*	*2*	−*34*	−*48*	−*22*	−*20*	−*96*	*5*
50	−61	−26	53	−72	−6	−34	−48	−22	**−6**	**−95**	**11**
45	−56	−22	53	−78	3				−6	−96	6
40	−50	−16	47	−79	−9				−23	−102	2
40	−58	−21	47	−85	−2				−25	−96	8
36	−31	−29	39	−88	12				−41	−89	−4
33	−63	−17	29	−93	14						
29	−52	−13									
27	−53	−14									
24	−70	−14									

The MNI coordinates from each of the 21 body-selective electrodes are grouped according to which cluster they were assigned to by the k-means clustering analysis. The MNI coordinates in the first row are the centroid locations within each cluster. Coordinates in bold typeface indicate an electrode that was body-specific as well as body-selective (see Materials and Methods).

The latency medians of faces, eyes, bodies, and flowers were 181, 195, 182, and 197 ms, respectively. The Wilcoxon signed rank test showed that the body response did not differ from the latency of the response to faces, *Z* = 1.40, *p* = 0.161, or eyes, *Z* = 0.83, *p* = 0.406. The latency of the peak to faces was marginally earlier than eyes, *Z* = 2.45, *p* = 0.014, but did not differ from flowers, *Z* = 1.74, *p* = 0.083. Eyes and flowers did not differ, *Z* = 0.12, *p* = 0.906.

The cluster detection algorithm identified two spatial clusters of electrodes within each hemisphere for eye-selective ERPs. The cluster centroids in the right hemisphere were located at 36, −55, 19 and 45, −83, −2. The cluster centroids in the left hemisphere were located at −34, −48, −22 and −20, −96, 5 (Figure 5). The small sample sizes within each cluster precluded statistical analysis of the ERPs. We describe the relevant qualitative results from within these clusters in the context of our Discussion Section.

In a second analysis, we identified body-*specific*, rather than body-*selective* (see Materials and Methods), electrodes. We identified only one body-specific site, which was located in the left hemisphere (bolded coordinates in Table 3).

### Voltage changes over space

We visually identified 10 occurrences from seven patients in which the peak of the ERP to all, or some, of the categories of interest changed at different rates across neighboring electrodes (Figure 4). Of these 10, seven were located on the ventral surface (6 right hemisphere) and three on the lateral occipitotemporal surface (1 right hemisphere). At many of these locations the most notable difference was between bodies and the other conditions. However, the peak rate of change between faces and eyes also differed, though this difference was often more subtle than that of bodies.

### Event-related spectral perturbations

Across the electrodes that were selective for faces (*N* = 32), eyes (*N* = 51), and bodies (*N* = 21), we observed substantial change in gamma power as an effect of stimulus presentation. However, there were few differences between categories. At the face-selective ERP locations the face-γERSP was larger than the eye-γERSP, *t*_(10)_ = 3.69, *p* = 0.004, and marginally larger than the body-γERSP, *t*_(10)_ = 2.46, *p* = 0.03. There were no other pairwise differences, *p*s > 0.01. At the eye-selective ERP locations eye-γERSP was *smaller* than the face-γERSP, *t*_(11)_ = 3.12, *p* = 0.010. This same relationship was seen even when only including face-specific sites in the eye-γERSP average. At these sites the face-γERSP was also marginally larger than the body γERSP, *t*_(11)_ = 2.67, *p* = 0.022. There were no other pairwise differences, *p*s >0.01. At the body-selective ERP locations there were no pairwise differences between conditions, *p*s >0.01.

We also investigated the category-selective γERSPs within each of the four spatial clusters identified by the cluster detection algorithm. As with the ERPs, the small samples within each cluster precluded statistical analyses. We describe the relevant qualitative results from within these clusters in the context of our discussion section.

## Discussion

In this paper we report several findings regarding the selectivity and organization of cortical areas engaged in the perception of faces, eyes, and bodies. Overall, we found substantial overlap in the activation by the three corporeal stimuli. The majority of electrodes selective for one of the three corporeal categories were selective for one or both of the other categories. However, we did identify several electrode sites that were specific for only a single category—particularly for eyes. Furthermore, we report evidence for different spatial distribution of voltage evoked by the three corporeal stimuli at closely adjacent electrodes. This indicates that the ERPs evoked by these stimuli are being generated by different configurations of current sinks and sources despite their substantial spatial overlap. In the following section we will discuss the evidence for this as well as general observations regarding the response properties and locations of face, eye, and body selective sites. We conclude by discussing these findings in the context of the perception of social agents.

### Neural selectivity of responses

Locations showing selectivity for at least one of the categories of interest were found widely distributed across bilateral VOTC. No category-selective locations were found in the frontal or parietal lobes. The wide spatial distribution observed in VOTC is consistent with prior iEEG reports from our laboratory (e.g., Allison et al., 1994a, 1999; Engell and McCarthy, 2010, 2011), but inconsistent with fMRI studies that often report highly localized selective regions (e.g., Sergent et al., 1992; Haxby et al., 1994; Puce et al., 1995; Kanwisher et al., 1997; Gauthier et al., 2000; but see Pinsk et al., 2009; Weiner and Grill-Spector, 2013). We used a cluster detection algorithm to assign each category-selective electrode to one of four spatial clusters, and then identified the centroids of those clusters. For each of the social agent conditions, the centroids (Figure 5) were well aligned to face- and body-selective areas identified in the fMRI literature. Face-selective electrodes clustered around the FFA (ventral temporal cortex) and OFA (posterior and lateral occipitotemporal cortex) in each hemisphere, as did eye- and body-selective electrodes. These findings suggest that group analysis of fMRI data has emphasized regions of maximal overlap at the expense of detecting spatial variability across, and perhaps within, individuals. Weiner and Grill-Spector (2013) have recently reported that high-resolution fMRI of individual participants shows that face- and body-selective regions repeat throughout the occipitotemporal cortex. If so, our findings might reflect a coarse sampling of this repeating pattern.

#### Face-selectivity

Category-selective face-N200s were identified at 32 locations across 11 patients. Consistent with a prior report (McCarthy et al., 1999), the peak of the face-N200 at these sites was qualitatively larger and earlier than the peak of the eye-N200. The face-N200 was also larger than the body-N200. Despite being selected for their face-selectivity, the body-N200 and eye-N200 at these face-selective sites was greater than the ERP evoked by flowers. Therefore, the underlying cortex is sensitive to non-face images of social agents, despite being optimally activated by faces. Notably though, the locations of the face-selective sites were widely distributed across bilateral VOTC and thus might span functionally heterogeneous regions that include some areas, such as the FFA located in right ventral temporal cortex (rVT), that are more face-selective than others.

Of the 32 face-selective electrodes, 14 were located within the rVT region, suggesting that there might indeed be greater face-selectivity within this region. However, the responses at these sites did not appreciably differ from those in our other three regions of interest. Perhaps more surprising was that none of the 14 face-*selective* sites within the rVT met the criteria to be face-*specific*. In other words, no electrodes on or around the FFA, a region often considered to be a functional module for face perception, were category-selective for faces as compared to eyes and/or bodies.

#### Eye-selectivity

Eyes are a critical feature of faces and attract the most attention during natural looking (Janik et al., 1978). In a previous iEEG study, McCarthy et al. (1999) found right VOTC and left LOTC sites at which face-parts (eyes, lips, noses) evoked a larger and later N200 than did whole faces. In that study, the authors averaged over the potentials independently evoked by eyes, lips, and noses. This approach creates the possibility that averaging in the potentially weaker responses of lips and noses will obscure eye-selective electrodes. Similarly, they averaged hands and flowers to create their control condition, and thus did not directly contrast face-parts with non-face body parts. In the current study, we focused on eyes and identified 51 eye-selective sites across twelve patients. The grand-averaged response to faces, eyes, and bodies across these sites was very similar to the response from face-selective sites, with the exception that the eye response was qualitatively larger and later than the face response. As with the face-selective sites, the body response at these eye-selective sites was larger (though not significantly so) than the flower response.

Unlike the face-selective sites, the eye-selective sites were more likely to also be category-specific. That is, 27 of the 51 sites were not identified as being selective for faces or bodies. Moreover, 13 of those 27 sites were eye-specific, all but two of which were located in bilateral occipital and lateral occipitotemporal cortex.

#### Body-selectivity

Category-selective body-N200s were identified at 21 electrodes across ten patients. As with the face-selective sites discussed above, the body-selective sites were also sensitive to the other social agent categories. All three categories were larger than flowers at these sites, and all were highly similar to one another. There was only a single electrode site in a single subject at which the body-N200 was category-specific. In other words, we found no evidence of regions that preferred bodies to faces or eyes. Particularly striking was the response of the subgroup of electrodes from within the right ventral temporal cortex, which includes the so-called fusiform body area (Schwarzlose et al., 2005). Here, the peak ERP response to bodies was qualitatively *smaller* than to faces or eyes (see Figure 5). The body-γERSP was also smaller than the face-γERSP. This contrasts to sites within the right occipitotemporal region (near the so-called extrastriate body area) where bodies elicited a larger N200 and γERSP than faces or eyes.

### Independent or shared neural substrate

As discussed above, neuroimaging studies report substantial overlap in VOTC brain regions activated by faces and bodies. The striking overlap of these networks is highlighted in a large-sample fMRI study (Engell and McCarthy, 2013). That study used dynamic “point light” displays (i.e., biological motion) rather than static body images, but the location and magnitude of activation evoked by bodies and point-light displays are strongly correlated (Peelen et al., 2006).

Our current ERP results offer some insight into the nature of this extensive overlap observed in fMRI. Consistent with fMRI studies, we found that electrodes sensitive to one visual category of animate agent (faces, eyes, or bodies) were frequently sensitive to one or both of the other categories. However, we found ten instances in which the amplitude of the peak ERP response changed at different rates across electrodes for two or more of the categories at closely adjacent electrodes. For instance, we observed a large difference in the rate of change for faces and for bodies (and to a lesser extent, eyes) across adjacent electrodes located on the right fusiform gyrus of Patient 1E (see Figure 4). Indeed, in this particular case the amplitude of the peak response increases for one condition while decreasing for the other. This differential rate of change in voltage across adjacent electrodes cannot be accounted for by a consistent configuration of current sources and sinks that is activated at different strengths. Rather it indicates that these ERP distributions are caused by a different pattern of input and/or the participation of at least some different neural elements for faces, bodies, and eyes.

It is important to note that sites at which *no* evidence was found for the different spatial rates of change for faces, bodies, and eyes outnumbered sites at which we found such evidence. However, a favorable spatial relationship between electrode locations and sink/source configurations is necessary to record category-selective N200s at neighboring electrodes. Therefore, an inability to find appropriate electrodes can be due simply to the unsystematic spatial sampling typical of subdural recordings. The presence of functionally heterogeneous, but spatially regular and interdigitated neurons at a scale much smaller than our inter-electrode distance could also result in indistinguishable voltage distributions with our methods. In contrast, we cannot think of alternative explanations that would account for differential rate change, thus making the ten instances across seven patients reported here compelling.

### Faces: part and whole

An influential model of face processing posits that detection and representation of face-parts occurs in the “occipital face area” of the posterior occipitotemporal cortex (Haxby et al., 2001). Consistent with this model, we found the majority of eye-specific sites in bilateral posterior occipitotemporal cortex. In addition, there were few face-selective sites in this region. However, our data is inconsistent with another key feature of this model; namely, that the fusiform face area is primarily involved in holistic processing of the whole face. Eye-selective sites in this region slightly outnumbered face-selective sites. Moreover, we found no electrodes in this region that were face-specific when compared to isolated eyes and bodies.

We have previously shown that the face-N200 is functionally distinct from the face-γERSP (Engell and McCarthy, 2010, 2011) and have proposed that the former is involved in early detection, whereas the latter is involved in elaborative processing. It is notable then, that the face-γERSP was *larger* than the eye-γERSP at eye-selective ERP sites. The qualitative nature of these results demand caution, but we speculate that the elaborative processing of a whole face follows the initial evoked response to eyes. This would reconcile the current results with the neuroimaging literature because changes in gamma power, and not evoked-potentials, are most closely related to changes in the hemodynamic response (Mukamel et al., 2005; Niessing et al., 2005; Lachaux et al., 2007; Koch et al., 2009; Ojemann et al., 2010; Scheeringa et al., 2011; Hermes et al., 2012). However, we observed that the face-γERSP is larger than the eye-γERSP at eye-selective sites within the OFA region as well, which is inconsistent with fMRI reports of a greater OFA response to face-parts(Liu et al., 2010).

## Conclusions

Direct electrical recordings from the surface of the fusiform gyrus and adjacent VOTC and LOTC show a complex pattern of activation for the perception of faces, bodies, and eyes. Most electrodes selective for one category of corporeal stimuli (relative to the control category of flowers) showed selectivity for the other corporeal categories as well. This was particularly true for body stimuli—only one electrode site of 1536 total sites examined, and of 69 sites showing a response selective for at least one category of corporeal stimuli, was specific for bodies. Perhaps most surprisingly, no electrode site in the vicinity of the fusiform face and body areas (as defined by fMRI studies) showed face or body specificity. These data do not, then, provide evidence for highly discrete processing regions for these different stimulus types. However, we did find a differential spatial distribution over closely adjacent electrodes for the maximum ERP response to bodies (and to a lesser extent for eyes) relative to faces. This suggests that *within* a given region, the different stimulus types engaged different configurations of current sinks and sources. Taken together, these results suggest a lumpy or patchy spatial representation for these different types of corporeal stimuli rather than segregation into highly discrete regions.

### Conflict of interest statement

The authors declare that the research was conducted in the absence of any commercial or financial relationships that could be construed as a potential conflict of interest.

## References

[B1] Allison T. Ginter H. McCarthy G. Nobre A. C. Puce A. Luby M. (1994a). Face recognition in human extrastriate cortex. J. Neurophysiol. 71, 821–825 8176446 10.1152/jn.1994.71.2.821

[B2] Allison T. McCarthy G. Nobre A. Puce A. Belger A. (1994b). Human extrastriate visual cortex and the perception of faces, words, numbers, and colors. Cereb. Cortex 4, 544–554 10.1093/cercor/4.5.544 7833655

[B3] Allison T. Puce A. Spencer D. D. McCarthy G. (1999). Electrophysiological studies of human face perception. I: Potentials generated in occipitotemporal cortex by face and non-face stimuli. Cereb. Cortex 9, 415–430 10.1093/cercor/9.5.415 10450888

[B4] Baylis G. C. Rolls E. T. Leonard C. M. (1985). Selectivity between faces in the responses of a population of neurons in the cortex in the superior temporal sulcus of the monkey. Brain Res. 342, 91–102 10.1016/0006-8993(85)91356-3 4041820

[B5] Bonda E. Petrides M. Ostry D. Evans A. (1996). Specific involvement of human parietal systems and the amygdala in the perception of biological motion. J. Neurosci. 16, 3737–3744 8642416 10.1523/JNEUROSCI.16-11-03737.1996 PMC6578830

[B6] Bracci S. Ietswaart M. Peelen M. V. Cavina-Pratesi C. (2010). Dissociable neural responses to hands and non-hand body parts in human left extrastriate visual cortex. J. Neurophysiol. 103, 3389–3397 10.1152/jn.00215.2010 20393066 PMC2888254

[B7] Calder A. J. Young A. W. (2005). Understanding the recognition of facial identity and facial expression. Nat. Rev. Neurosci. 6, 641–651 10.1038/nrn1724 16062171

[B8] de Gelder B. Van den Stock J. Meeren H. K. Sinke C. B. Kret M. E. Tamietto M. (2010). Standing up for the body. Recent progress in uncovering the networks involved in the perception of bodies and bodily expressions. Neurosci. Biobehav. Rev. 34, 513–527 10.1016/j.neubiorev.2009.10.008 19857515

[B9] Delorme A. Makeig S. (2004). EEGLAB: an open source toolbox for analysis of single-trial EEG dynamics including independent component analysis. J. Neurosci. Methods 134, 9–21 10.1016/j.jneumeth.2003.10.009 15102499

[B10] Downing P. E. Peelen M. V. (2011). The role of occipitotemporal body-selective regions in person perception. Cogn. Neurosci. 2, 186–203 10.1080/17588928.2011.582945 24168534

[B11] Engell A. D. Huettel S. McCarthy G. (2012). The fMRI BOLD signal tracks electrophysiological spectral perturbations, not event-related potentials. Neuroimage 59, 2600–2606 10.1016/j.neuroimage.2011.08.079 21925278 PMC3277784

[B12] Engell A. D. McCarthy G. (2010). Selective attention modulates face-specific induced gamma oscillations recorded from ventral occipitotemporal cortex. J. Neurosci. 30, 8780–8786 10.1523/JNEUROSCI.1575-10.2010 20592199 PMC2918280

[B13] Engell A. D. McCarthy G. (2011). The relationship of gamma oscillations and face-specific ERPs recorded subdurally from occipitotemporal cortex. Cereb. Cortex 21, 1213–1221 10.1093/cercor/bhq206 20961973 PMC3077434

[B14] Engell A. D. McCarthy G. (2013). Probabilistic atlases for face and biological motion perception: an analysis of their reliability and overlap. Neuroimage 74, 140–151 10.1016/j.neuroimage.2013.02.025 23435213 PMC3690657

[B15] Engell A. D. McCarthy G. (2014). Repetition suppression of face-selective evoked and induced EEG recorded from human cortex. Hum. Brain Mapp. 35, 4155–4162 10.1002/hbm.22467 24677530 PMC4322774

[B16] Fisch L. Privman E. Ramot M. Harel M. Nir Y. Kipervasser S. (2009). Neural “ignition”: enhanced activation linked to perceptual awareness in human ventral stream visual cortex. Neuron 64, 562–574 10.1016/j.neuron.2009.11.001 19945397 PMC2854160

[B17] Gauthier I. Tarr M. J. Moylan J. Skudlarski P. Gore J. C. Anderson A. W. (2000). The fusiform face area is part of a network that processes faces at the individual level. J. Cogn. Neurosci. 12, 495–504 10.1162/089892900562165 10931774

[B18] Grezes J. Pichon S. De Gelder B. (2007). Perceiving fear in dynamic body expressions. Neuroimage 35, 959–967 10.1016/j.neuroimage.2006.11.030 17270466

[B19] Gross C. G. Bender D. B. Rocha-Miranda C. E. (1969). Visual receptive fields of neurons in inferotemporal cortex of the monkey. Science 166, 1303–1306 10.1126/science.166.3910.1303 4982685

[B20] Gross C. G. Rocha-Miranda C. E. Bender D. B. (1972). Visual properties of neurons in inferotemporal cortex of the macaque. J. Neurophysiol. 35, 96–111 4621506 10.1152/jn.1972.35.1.96

[B21] Grossman E. D. Blake R. (2002). Brain areas active during visual perception of biological motion. Neuron 35, 1167–1175 10.1016/S0896-6273(02)00897-8 12354405

[B22] Haxby J. V. Gobbini M. I. Furey M. L. Ishai A. Schouten J. L. Pietrini P. (2001). Distributed and overlapping representations of faces and objects in ventral temporal cortex. Science 293, 2425–2430 10.1126/science.1063736 11577229

[B23] Haxby J. V. Hoffman E. A. Gobbini M. I. (2000). The distributed human neural system for face perception. Trends Cogn. Sci. 4, 223–233 10.1016/S1364-6613(00)01482-0 10827445

[B24] Haxby J. V. Horwitz B. Ungerleider L. G. Maisog J. M. Pietrini P. Grady C. L. (1994). The functional organization of human extrastriate cortex: a PET-rCBF study of selective attention to faces and locations. J. Neurosci. 14, 6336–6353 7965040 10.1523/JNEUROSCI.14-11-06336.1994 PMC6577268

[B25] Hermes D. Miller K. J. Vansteensel M. J. Aarnoutse E. J. Leijten F. S. Ramsey N. F. (2012). Neurophysiologic correlates of fMRI in human motor cortex. Hum. Brain Mapp. 33, 1689–1699 10.1002/hbm.21314 21692146 PMC6870225

[B26] Ishai A. (2008). Let's face it: it's a cortical network. Neuroimage 40, 415–419 10.1016/j.neuroimage.2007.10.040 18063389

[B27] Janik S. W. Wellens A. R. Goldberg M. L. DellOsso L. F. (1978). Eyes as the center of focus in the visual examination of human faces. Percept. Mot. Skills 47, 857–858 10.2466/pms.1978.47.3.857 740480

[B28] Kanwisher N. McDermott J. Chun M. M. (1997). The fusiform face area: a module in human extrastriate cortex specialized for face perception. J. Neurosci. 17, 4302–4311 9151747 10.1523/JNEUROSCI.17-11-04302.1997 PMC6573547

[B29] Koch S. P. Werner P. Steinbrink J. Fries P. Obrig H. (2009). Stimulus-induced and state-dependent sustained gamma activity is tightly coupled to the hemodynamic response in humans. J. Neurosci. 29, 13962–13970 10.1523/JNEUROSCI.1402-09.2009 19890006 PMC6666720

[B30] Lachaux J. P. Fonlupt P. Kahane P. Minotti L. Hoffmann D. Bertrand O. (2007). Relationship between task-related gamma oscillations and BOLD signal: new insights from combined fMRI and intracranial EEG. Hum. Brain Mapp. 28, 1368–1375 10.1002/hbm.20352 17274021 PMC6871347

[B31] Lachaux J. P. George N. Tallon-Baudry C. Martinerie J. Hugueville L. Minotti L. (2005). The many faces of the gamma band response to complex visual stimuli. Neuroimage 25, 491–501 10.1016/j.neuroimage.2004.11.052 15784428

[B32] Leonard C. M. Rolls E. T. Wilson F. A. W. Baylis G. C. (1985). Neurons in the amygdala of the monkey with responses selective for faces. Behav. Brain Res. 15, 159–176 10.1016/0166-4328(85)90062-2 3994832

[B33] Liu J. Harris A. Kanwisher N. (2010). Perception of face parts and face configurations: an fMRI study. J. Cogn. Neurosci. 22, 203–211 10.1162/jocn.2009.21203 19302006 PMC2888696

[B34] McCarthy G. Puce A. Belger A. Allison T. (1999). Electrophysiological studies of human face perception. II: Response properties of face-specific potentials generated in occipitotemporal cortex. Cereb. Cortex 9, 431–444 10.1093/cercor/9.5.431 10450889

[B35] Mukamel R. Gelbard H. Arieli A. Hasson U. Fried I. Malach R. (2005). Coupling between neuronal firing, field potentials, and FMRI in human auditory cortex. Science 309, 951–954 10.1126/science.1110913 16081741

[B36] Niessing J. Ebisch B. Schmidt K. E. Niessing M. Singer W. Galuske R. A. (2005). Hemodynamic signals correlate tightly with synchronized gamma oscillations. Science 309, 948–951 10.1126/science.1110948 16081740

[B37] Ojemann G. A. Corina D. P. Corrigan N. Schoenfield-McNeill J. Poliakov A. Zamora L. (2010). Neuronal correlates of functional magnetic resonance imaging in human temporal cortex. Brain 133, 46–59 10.1093/brain/awp227 19773355 PMC2801320

[B38] Peelen M. V. Downing P. E. (2005). Selectivity for the human body in the fusiform gyrus. J. Neurophysiol. 93, 603–608 10.1152/jn.00513.2004 15295012

[B39] Peelen M. V. Wiggett A. J. Downing P. E. (2006). Patterns of fMRI activity dissociate overlapping functional brain areas that respond to biological motion. Neuron 49, 815–822 10.1016/j.neuron.2006.02.004 16543130

[B40] Perrett D. I. Rolls E. T. Caan W. (1982). Visual neurones responsive to faces in the monkey temporal cortex. Exp. Brain Res. 47, 329–342 10.1007/BF00239352 7128705

[B41] Pichon S. de Gelder B. Grèzes J. (2008). Emotional modulation of visual and motor areas by dynamic body expressions of anger. Soc. Neurosci. 3, 199–212 10.1080/17470910701394368 18979376

[B42] Pinsk M. A. Arcaro M. Weiner K. S. Kalkus J. F. Inati S. J. Gross C. G. (2009). Neural representations of faces and body parts in macaque and human cortex: a comparative FMRI study. J. Neurophysiol. 101, 2581–2600 10.1152/jn.91198.2008 19225169 PMC2681436

[B43] Pitcher D. Walsh V. Duchaine B. (2011). The role of the occipital face area in the cortical face perception network. Exp. Brain Res. 209, 481–493 10.1007/s00221-011-2579-1 21318346

[B44] Pourtois G. Peelen M. V. Spinelli L. Seeck M. Vuilleumier P. (2007). Direct intracranial recording of body-selective responses in human extrastriate visual cortex. Neuropsychologia 45, 2621–2625 10.1016/j.neuropsychologia.2007.04.005 17499819

[B45] Puce A. Allison T. Gore J. C. McCarthy G. (1995). Face-sensitive regions in human extrastriate cortex studied by functional MRI. J. Neurophysiol. 74, 1192–1199 7500143 10.1152/jn.1995.74.3.1192

[B46] Puce A. Allison T. McCarthy G. (1999). Electrophysiological studies of human face perception. III: Effects of top-down processing on face-specific potentials. Cereb. Cortex 9, 445–458 10.1093/cercor/9.5.445 10450890

[B47] Puce A. Allison T. Spencer S. S. Spencer D. D. McCarthy G. (1997). Comparison of cortical activation evoked by faces measured by intracranial field potentials and functional MRI: two case studies. Hum. Brain Mapp. 5, 298–305 20408232 10.1002/(SICI)1097-0193(1997)5:4<298::AID-HBM16>3.0.CO;2-A

[B48] Rossion B. Caldara R. Seghier M. Schuller A. M. Lazeyras F. Mayer E. (2003). A network of occipito-temporal face-sensitive areas besides the right middle fusiform gyrus is necessary for normal face processing. Brain 126, 2381–2395 10.1093/brain/awg241 12876150

[B49] Scheeringa R. Fries P. Petersson K.-M. Oostenveld R. Grothe I. Norris D. G. (2011). Neuronal dynamics underlying high-and low-frequency EEG oscillations contribute independently to the human BOLD signal. Neuron 69, 572–583 10.1016/j.neuron.2010.11.044 21315266

[B50] Schwarzlose R. F. Baker C. I. Kanwisher N. (2005). Separate face and body selectivity on the fusiform gyrus. J. Neurosci. 25, 11055–11059 10.1523/JNEUROSCI.2621-05.2005 16306418 PMC6725864

[B51] Sergent J. Ohta S. MacDonald B. (1992). Functional neuroanatomy of face and object processing. A positron emission tomography study. Brain 115(Pt 1), 15–36 10.1093/brain/115.1.15 1559150

[B52] Shultz S. McCarthy G. (2012). Goal-directed actions activate the face-sensitive posterior superior temporal sulcus and fusiform gyrus in the absence of human-like perceptual cues. Cereb. Cortex 22, 1098–1106 10.1093/cercor/bhr180 21768227 PMC3450923

[B53] Shultz S. van den Honert R. N. Engell A. D. McCarthy G. (2014). Stimulus-induced reversal of information flow through a cortical network for animacy perception. Soc. Cogn. Affect. Neurosci. [Epub ahead of print]. 10.1093/scan/nsu028 24625785 PMC4994845

[B54] Singer W. Gray C. M. (1995). Visual feature integration and the temporal correlation hypothesis. Annu. Rev. Neurosci. 18, 555–586 10.1146/annurev.ne.18.030195.003011 7605074

[B55] Spencer S. S. Spencer D. D. Williamson P. D. Mattson R. H. (1982). The localizing value of depth electroencephalography in 32 patients with refractory epilepsy. Ann. Neurol. 12, 248–253 10.1002/ana.410120306 6814350

[B56] Tallon-Baudry C. Bertrand O. (1999). Oscillatory gamma activity in humans and its role in object representation. Trends Cogn. Sci. 3, 151–162 10.1016/S1364-6613(99)01299-1 10322469

[B57] Tsuchiya N. Kawasaki H. Oya H. Howard M. A. Adolphs R. (2008). Decoding face information in time, frequency and space from direct intracranial recordings of the human brain. PLoS ONE 3:e3892 10.1371/journal.pone.0003892 19065268 PMC2588533

[B58] van de Riet W. A. Grezes J. de Gelder B. (2009). Specific and common brain regions involved in the perception of faces and bodies and the representation of their emotional expressions. Soc. Neurosci. 4, 101–120 10.1080/17470910701865367 19255912

[B59] Weiner K. S. Grill-Spector K. (2013). Neural representations of faces and limbs neighbor in human high-level visual cortex: evidence for a new organization principle. Psychol. Res. 77, 74–97 10.1007/s00426-011-0392-x 22139022 PMC3535411

